# ‘It opened my eyes’: Parents’ experiences of their child receiving an anxiety disorder diagnosis

**DOI:** 10.1177/13591045221088708

**Published:** 2022-04-25

**Authors:** Emily Davey, Cathy Creswell, Ray Percy, Tessa Reardon

**Affiliations:** 1 Departments of Experimental Psychology and Psychiatry, 6396 University of Oxford, Oxford, UK; 2 School of Psychology and Clinical Language Sciences, 6816 University of Reading, Reading, UK

**Keywords:** Anxiety disorder, diagnosis, children, parents, qualitative research

## Abstract

Anxiety disorders are the most common mental health disorders among children, however there is limited guidance on the process of assessing child anxiety disorders and sharing diagnostic outcomes with families. This study aimed to identify aspects of the diagnostic process that are helpful and/or unhelpful for families, and ways to mitigate any potential negative consequences of receiving a child anxiety disorder diagnosis. A qualitative study was conducted with parents of 11 children (aged 7–12 years) with a primary diagnosis of an anxiety disorder, identified through a child mental health service. We used an inductive thematic analysis approach. Elements of the diagnostic process considered helpful or less helpful for families related to four themes: clarity and insight, being heard, the anxiety label and access to support. Findings illustrate the importance of sharing diagnoses compassionately in the context of ensuing treatment, and the benefits of providing families with personalised verbal and written diagnostic information, that is tailored for both parents and children.

## Introduction

The diagnosis of mental health problems attracts considerable debate. Mental health diagnoses can explain symptoms and facilitate access to suitable intervention but may also have unintended negative consequences (Craddock & Mynors-Wallis, 2014). There are particular concerns that mental health diagnoses in children may medicalise emotional and behavioural problems which occur as part of normal development (Frances & Batstra, 2013), and health professionals may be reluctant to diagnose a child with a mental health disorder due to stigma (Shafran et al., 2018). Despite these concerns, there is growing consensus that we need to improve early identification and prompt access to evidence-based interventions for common mental health problems in children (e.g. Department of Health and Social Care & Department for Education, 2017). Evidence-based interventions typically target specific mental health disorders in children so accurate identification and diagnosis of a mental health problem is a critical first step to accessing appropriate intervention and specialist support (Shafran et al., 2018).

There is a lack of guidance regarding the process of using diagnoses for common mental health problems in children, and clinicians report uncertainty about how to share these diagnoses with families (O’Brien et al., 2017). National Institute for Health and Care Excellence (NICE) guidance specifically addresses the diagnosis of neurodevelopmental disorders in children and provides recommendations on the process of assigning Autism Spectrum Disorder (ASD) and Attention Deficit Hyperactivity Disorder (ADHD) diagnoses in children and communicating these diagnoses with families (NICE, 2017; NICE, 2019). However, corresponding NICE guidance for mental health disorders that occur across the lifespan do not address the diagnostic process specifically in children. Anxiety disorders are the most common mental health disorders among children, with an average age of onset of 11 years (Polanczyk et al., 2015; Kessler et al., 2005), but there is limited guidance on the process of assessing anxiety disorders in children. NICE guidelines address the assessment of social anxiety disorder in children and provide recommendations on the content of a comprehensive assessment and tools to aid identification (NICE, 2013), but do not address other anxiety disorders or the process of sharing diagnoses with families.

Gaining insight into individuals’ experiences of the diagnostic process and receiving a specific diagnosis is critical to inform guidance on how to assign specific mental health diagnoses. The importance of incorporating service users’ views into the design and delivery of services and the benefits of using qualitative research to generate detailed insight into experiences of services are widely recognised (Department of Health, 2004; Hammarberg et al., 2016). In the context of diagnosing mental health disorders in children, it is particularly critical to understand parents’ experiences. Children with mental health problems are reliant on their parents to seek help on their behalf, and parents typically act as liaisons between professionals and children (Bringewatt, 2017). Consequently, parents’ experiences of the process of seeking help for their child and their child receiving a diagnosis may shape the family’s subsequent engagement with child mental health services (McKay & Bannon, 2004).

Qualitative studies examining parents’ experiences of their child receiving a diagnosis of ASD or ADHD illustrate the potential benefits of a diagnosis, as well as parental concerns related to the diagnostic process. Parents often perceive their child’s diagnosis as simultaneously empowering and stigmatising (Makino et al., 2021; Ringer et al., 2020). Whilst a diagnosis can offer families a framework to understand their experiences, vindicate their concerns and facilitate access to support (e.g. Makino et al., 2021), it can also provoke concerns regarding stigma, their child’s future, and disclosing the diagnosis to others (e.g. Eaton et al., 2017; Ringer et al., 2020). Parents’ experiences of seeking support for their child can shape their experience of the diagnostic process, with those who experience significant delays and disappointment with available support expressing frustration and anger (Makino et al., 2021). In contrast, receiving clear diagnostic information that is easy to understand and shared sensitively seems to enhance parents’ satisfaction with the process (e.g. Abbott et al., 2013). These findings have clear implications for the use of neurodevelopmental disorder diagnoses, and how this information is shared with parents, but we do not know the extent to which these studies capture experiences that are relevant in the context of child anxiety disorder diagnoses.

There may be particular features of anxiety disorders and perceptions of anxiety treatments that influence parents’ experiences of their child receiving an anxiety diagnosis. For example, parents often face challenges differentiating between developmentally appropriate fears and worries and clinically significant anxiety problems (Reardon et al., 2018). This raises questions about whether parents’ confidence in their ability to identify their child’s anxiety difficulties may shape their experience of the diagnostic process and the extent to which they perceive it as helpful. Moreover, whilst neurodevelopmental disorders typically cause impairment into adulthood, child anxiety disorders can be effectively treated with psychological interventions (James et al., 2020), which may influence parents’ perceptions of child anxiety disorder diagnoses. An improved understanding of parents’ experiences of their child receiving an anxiety disorder diagnosis would inform guidance on the use of these specific diagnoses and help to ensure that any disorder-specific unintended negative consequences are minimised.

We sought to explore parents’ experiences of their child receiving an anxiety disorder diagnosis. Specifically, we aimed to identify how and why the diagnostic process was helpful and/or not helpful for families, and ways to mitigate any potential negative consequences of receiving an anxiety disorder diagnosis. Given that anxiety disorders typically first occur by age 12 years, and parents’ pivotal role in the help-seeking and diagnostic process for pre-adolescents, we focused on pre-adolescent children (aged 7–12 years). An inductive qualitative approach was adopted to cultivate a detailed insight into experiences described by parents.

## Method

The study was approved by the School of Psychology and Clinical Language Sciences Research Ethics Committee at the University of Reading (REC reference: 2018/030) and the National Research Ethics Service Committee South Central - Hampshire B (REC reference: 18/SC/0214). Consolidated criteria for reporting qualitative studies are followed (see Online Supplement 1).

### Participant selection

Participants were recruited via the Anxiety and Depression in Young People (AnDY) Research Clinic, a clinical service that receives referrals from primary and secondary care services and is funded by local NHS commissioning. All children who attended the clinic received a routine diagnostic assessment, and outcomes from these routine assessments were used to identify potential participants. Parents of children (aged 7–12 years), whose primary presenting disorder was a DSM-5 anxiety disorder, formed a pool of 48 potential participants. To help capture varied experiences of the diagnostic process, we used purposive sampling (Robinson, 2014) to select parents to invite from the pool of 48. We invited families that varied in relation to the following criteria: (i) child age and gender; (ii) type and severity of child anxiety disorder; and (iii) stage in the treatment process (before/during/after treatment).

We sent written study information to 33 parents, and one week later attempted to contact all of these parents by telephone. We verbally invited 21 (12 could not be reached by telephone), and interviews were conducted with 11 parents (5 agreed but did not attend; 5 declined). Participants provided written informed consent. Recruitment continued until analyses indicated that the dataset was sufficiently rich and varied to address study aims (Braun & Clarke, 2021). One-to-one interviews were conducted with mothers of 6 girls and 5 boys, aged 7–12 years (median age 10 years). A variety of primary anxiety disorders were included in the sample, including separation anxiety disorder (*n* = 1), social anxiety disorder (*n =* 2), specific phobia (*n =* 4), generalised anxiety disorder (GAD; *n* = 3) and unspecified anxiety disorder (*n* = 1), with clinical severity ratings ranging from 4 to 7. There was also variation in families’ stage in the treatment process (before, *n* = 3; during, *n* = 4; after, *n* = 4).

### Procedure

#### Routine diagnostic assessment

Each diagnostic assessment included separate child and parent interviews, administered by research assistants (Psychology graduates) trained to a high level of inter-rater reliability (see Online Supplement 2 for full details of diagnostic assessment).

Following the diagnostic assessment, families attended a routine follow-up appointment to discuss assessment outcomes and treatment options; children with a primary anxiety disorder were offered parent-led, therapist-guided, cognitive behavioural therapy (CBT). Parents were then sent a written report which summarised the assessment findings and diagnoses.

#### Qualitative interviews

The interviews were conducted by a female MSc student in Clinical Psychology (ED) from June 2018 to January 2019. One interview was conducted face-to-face and 10 were completed by telephone, with an average duration of 32 minutes. The development of the interview topic-guide was informed by relevant literature and consultation with the AnDY Research Advisory Group. Interviews encouraged discussion around (i) experiences of the routine diagnostic process; (ii) emotional response to the diagnosis; (iii) experiences of sharing the diagnosis with others; and (iv) perceived relevance of the diagnosis (see Online Supplement 3). The topic-guide was used flexibly, enabling the researcher to adapt or generate questions in response to participants’ answers and field notes were collected. All interviews were audio-recorded and transcribed verbatim, with identifiable information removed and participants’ names replaced with pseudonyms.

### Analysis

A reflexive, inductive thematic analysis approach was used to code and interpret the data (Braun & Clarke, 2006), with codes and themes derived from the data. *NVivo* was used to facilitate coding. Codes were generated through an iterative process, whereby earlier transcripts were reviewed and refined as each new transcript was coded. Similar codes were clustered together to enable potential themes to emerge from the data. The analysis was led by one researcher (ED) who met regularly with team members (TR and CC) to discuss codes and emerging themes, allowing for alternative interpretations of the data. Further refinement of emerging themes aided clear boundaries for the final themes.

## Results

Findings are described in relation to four themes: (i) clarity and insight; (ii) being heard; (iii) anxiety label; and (iv) access to support. Elements of the diagnostic process considered helpful and/or less helpful for families were identified in relation to each of these overarching themes.

### Clarity and insight: ‘It opened my eyes’

Receiving the diagnosis helped parents and children to make sense of the child’s thoughts, feelings, and behaviours. Parents were appreciative of information specific to their child as it helped them to understand their *own* child’s presentation: ‘I can see if he’s clenching his fist or if he’s breathing differently, which was all identified from the interviews’. (Susan). Prior to the diagnosis, many parents felt that their child was misbehaving and after receiving the diagnosis, they were able to reattribute their child’s behaviour to the anxiety disorder: ‘[The diagnosis] opened my eyes more that it was more than her just being naughty or trying to get my attention’. (Sandra). The diagnosis also appeared to help some children reframe and externalise their difficulties to the disorder: ‘I think [my child] felt that what she was experiencing was her fault… having a diagnosis means “that’s not me, it’s the anxiety making me feel like that”’. (Teresa). For some families, the treatment process enhanced understanding gained from the diagnosis, with families who had completed treatment reflecting positively on the diagnosis: ‘Knowing now what to look out for, how to respond to him when he is clearly experiencing these episodes of anxiety’. (Susan). Parents also identified the diagnosis as a tool to help others understand their child’s behaviour: ‘If anybody comments on the fact she’s being a bit funny or whatever, it’s quite handy in a way to turn around and say “well you do realise that she has actually got social anxiety, she’s not just being a pain”’. (Teresa).

Parents’ understanding of their child’s difficulties prior to the diagnosis seemed to influence the clarity and insight gained from receiving the diagnosis. Some parents described how they lacked clarity regarding their child’s difficulties prior to diagnosis: ‘We were just sort of wandering around in the dark before really, not knowing what to do or how to deal with [child]’. (Jane). Lacking an explanation for their child’s difficulties was distressing and frustrating: ‘Looking at him every day and seeing him really sad, seeing him worried, and not knowing what was going on with him was awful’. (Mary), and the diagnosis provided these parents with an explanation. In contrast, other parents were confident in their understanding of their child’s behaviour prior to the diagnostic process. These parents spoke apathetically about their child’s diagnosis as it confirmed what they already ‘knew’ (Kirsty), with one parent referring to it as a ‘foregone conclusion’ (Denise). If parents felt that they already had a good understanding of their child’s difficulties, the diagnostic process caused frustration: ‘The whole thing was extremely drawn out and tedious because I already knew he had a [specific phobia]’. (Denise).

The amount and quality of information parents received about their child’s diagnosis influenced the clarity and insight gained. Parents sought clear and accessible information and commented on the mechanisms through which they received this information. Parents spoke positively about receiving a detailed description of their child’s diagnosis face-to-face: ‘It was really good, the way that they obviously explained it fully before just giving us a report with lots of writing on, because sometimes that doesn’t really make sense’. (Jane), and how this provided an opportunity to ask questions. Parents valued the clinician being sensitive in their disclosure of the diagnosis and allowing time to digest the information: ‘There was never any sense of drama or you should be really concerned about this, it was all very calm, very gentle, very clearly explained, which left us with nothing we felt like we needed to worry about’. (Mary). However, some parents cited the danger of ‘information overload’ during this appointment, and difficulty absorbing the verbal information: ‘When you’re given a diagnosis that’s just face-to-face, you just hear the diagnosis, you don’t hear anything else about it’. (Christine). Consequently, parents appreciated receiving written information to supplement their understanding: ‘It was very good to have all of that on paper, summarised… so that I didn’t forget’. (Mary).

The extent to which the diagnostic information was accessible to families influenced their perception of the diagnostic process. Whilst the majority of parents felt that the information was suitable for parents, some expressed concerns about the age-appropriateness of the information for their children. One parent suggested it may help to have two separate conversations about the diagnosis: ‘…maybe have a private chat without [child] there, and then to introduce her maybe and explain it in children’s terms what [the diagnosis] meant’. (Sandra). Another parent felt visual aids may be helpful for children: ‘Maybe just some pictures or something like that might be helpful just to help them understand’. (Kirsty).

### Being heard: ‘I’d shared, he’d shared, and we weren’t by ourselves anymore’

Parents valued the opportunity to share their experiences with a professional during the assessment. Separate parent and child interviews enabled families to be open and honest in their responses: ‘I think it probably allowed [child] to say a lot more than he would have if I was there’. (Mary); ‘It was actually quite nice to be away from [child] to be able to talk about how I felt as well with regards to her’. (Christine). Many families seemed to find talking to someone about their child’s difficulties therapeutic: ‘All in all, I started feeling more positive because I’d shared, he’d shared, and we weren’t by ourselves anymore’. (Susan). However, parents did vary in their views related to the comprehensiveness of the assessment. Whilst some parents felt it was necessary to have a thorough appraisal and appreciated the opportunity to share their experiences in detail, others felt there was an expectation to overshare: ‘Loads and loads of questions were extremely inappropriate and didn’t seem to be relevant’. (Denise).

Parents emphasised the importance of the family-clinician relationship established throughout the diagnostic process. Many parents described their clinician as non-judgemental and supportive: ‘We felt like we could speak very openly about obviously any issues that we were having with [child], ...no judgment was being made on us as parents’. (Jane). Parents were grateful that their experiences had been corroborated by a professional: ‘I’m glad that somebody actually listened and that we weren’t fobbed off and they took us seriously’. (Linda). One parent described how their child had been ‘empowered’ by the acknowledgement of her difficulties: ‘I think that has been the most important thing, that [child] has been listened to, that somebody who is in a medical authority position has listened to her and believed her’. (Christine).

### Anxiety label: ‘You’re stuck with it for life’

Parents’ perceptions of the anxiety disorder ‘label’ were influenced by the extent to which the diagnosis aligned with their experiences. Prior to the diagnostic process, parents varied in the extent to which they felt their child was experiencing difficulties with anxiety, and whether anxiety was their child’s primary difficulty or not. Parents who felt that an anxiety disorder ‘label’ accurately captured their child’s difficulties spoke about the diagnosis in positive terms: ‘I found it quite comforting in the end… actually what I thought it was, I was on the right track with her’. (Christine). Whereas, if the diagnosis did not correspond with parents’ beliefs about their child’s symptoms, their experience was more conflicted. In particular, parents who felt that their child’s difficulties had been overlooked were less assured on receipt of the diagnosis: ‘I was still concerned about the low mood’. (Mary). Notably, concerns regarding the accuracy of the diagnosis were particularly evident among parents of children whose primary diagnosis was GAD. One of these parents described how she thought her child had been misdiagnosed and that this led to inappropriate treatment: ‘I think we’ve still got some underlying issues that haven’t been addressed’. (Karen).

The perceived chronicity of anxiety disorders also seemed to shape parents’ perceptions of the anxiety ‘label’. Some parents viewed anxiety disorders as short-term conditions which can be readily treated: ‘This is what she’s currently struggling with, it’s totally recoverable from for want of a better word’. (Julie). Notably, however, some parents whose children had completed treatment believed that their child was going to experience anxiety into adulthood: ‘I can’t imagine it’s going to be “yay fine, she’s cured, she’s never going to suffer again”’ (Teresa). Nonetheless, when the clinician communicated optimism about the child’s future, parents found this supportive: ‘It’s just sort of given everyone a bit of reassurance that stuff is very positive and we can move forward’. (Jane).

Parents’ views related to the potentially pejorative consequences of ‘labelling’ appeared to influence their experiences of the diagnostic process. In particular, views surrounding the stigma of ‘labelling’ were important determinants of parents’ openness to share the diagnosis with others. Some parents spoke emphatically about the anxiety label and how the potential consequences for their child deterred them from disclosing the diagnosis with others: ‘The difficulty with the labelling is that everybody looks at her through those glasses and I don’t know that that’s beneficial’. (Julie). Other parents were unperturbed by the stigma of labels: ‘Mental health is nothing to be ashamed of… I wouldn’t be at all worried about telling anybody if they wanted to know’ (Christine). Some parents also had concerns that their child might over-identify with the anxiety label and misuse it as justification for other behaviours: ‘Sometimes you’re worried that she’s just using it as an excuse to be rude, to be nasty to everybody’. (Linda). Parents also expressed trepidation about the long-term impact: ‘This sense that that’s it – you’re stuck with it for life – which kind of in a way for me removes or puts inside her almost a sense of hopelessness that this is who she is’. (Julie). Another concern conveyed by some parents was the concept of diagnostic overshadowing; all behaviour being attributed to the anxiety disorder and other co-existing problems going unnoticed: ‘It’s easy then, isn’t it, to put all behaviour or any of the behaviour that might fit into that category down to anxiety and potentially miss other stuff that is going on’. (Julie).

Parents described mixed and transient emotional responses to their child’s anxiety disorder diagnosis. Some parents’ immediate reaction was a sense of relief and affirmation that their experience of their child’s difficulties had been validated: ‘A slight reassurance that he actually does have a phobia and it isn’t just me going crazy’. (Denise). Receipt of the diagnosis evoked more ambivalent feelings for other parents, with relief moderated by a loss of vision of a ‘normal’ child: ‘I was hoping they were going to say there was nothing really wrong with him’. (Mary). Similarly, some parents described experiencing feelings of guilt and sadness: ‘I felt sad. I felt [the anxiety] came from me’. (Susan); ‘Really upset for her… she’s just a little girl, you should be able to be a child for as long as you possibly can’. (Christine). Despite some ambivalent feelings, most parents viewed the diagnostic process as a constructive step towards receiving support, bringing attention to their child’s difficulties and giving them ‘something to work with’ (Mary). Reflecting on their child’s diagnosis, parents described how their feelings of apprehension and upset diminished over time: ‘I feel a lot more at ease and a lot happier now’. (Jane).

### Access to support: ‘The only way you can get help’

The extent to which parents felt that the diagnosis successfully prompted access to appropriate professional support was pivotal to their overall experience of their child’s diagnosis. Parents described how a definitive diagnosis was necessary for their child to procure treatment for their anxiety: ‘The only way you can get help is if you’re diagnosed with something’. (Linda). Some parents felt that the sole benefit of a diagnosis is that it facilitates access to treatment, and did not differentiate the anxiety disorder label from their child’s treatment: ‘I understand that she needs a diagnosis in order to justify being able to be seen at the clinic, but other than that… I don’t really see that it serves any purpose’. (Julie). Although, notably parents generally felt a diagnosis should not be a prerequisite to accessing services for their child’s anxiety: ‘The fact that she’d already been treated and diagnosed didn’t seem to be enough to be able to access treatment… which seems completely ludicrous to me’. (Julie). Some parents also expressed concern that the diagnosis only enabled access to treatment at one service, not longer-term support: ‘It’s just something that I’ve got in writing, it doesn’t mean anything… once treatment at AnDY stopped, it stopped’. (Linda).

In addition to gaining access to clinical treatment, access to additional forms of support resulting from the diagnosis was clearly important to families. Some parents spoke positively about how the diagnosis helped their child to access support at school: ‘A SENCO teacher at school… now works quite closely with [child], you know if he has kind of any of his meltdowns or his trouble in the morning with like separating from me’. (Kirsty). Whilst other parents expressed their dissatisfaction with the lack of support that their child had received at school: ‘The pastoral care team, not so much help as I thought that I’d get from them’. (Linda). For some parents, sharing their child’s diagnosis resulted in both them and their child receiving more support from family and friends: ‘My friend actually, her child went through a very similar situation so she has been massively supportive to us’. (Jane).

It was also clear that the diagnosis helped some families to provide support for their child. Equipped with an anxiety label for their child’s difficulties, parents described feeling more in control of their situation and better able to support their child: ‘You can do something about it when you know what it is, problem solve around it’. (Christine); ‘Knowing that she’s got a disorder and actually that she’s not doing something on purpose made me feel different about the way I approach some things’. (Sandra). In particular, parents explained how the ascription of an anxiety label encouraged them to be more empathic and less dismissive: ‘I think it just kind of helps you be a bit more empathic because you kind of just think how would someone that kind of suffers from anxiety kind of feel in this situation’. (Kirsty). Many parents described how the diagnostic process highlighted the importance of communication between them and their child and that an enhanced understanding of their child’s needs increased familial harmony: ‘Our relationships are better; my relationship with him, my husband’s relationship with him, and then things have improved with his sisters as well’. (Mary).

## Discussion

### Main findings

In-depth analysis of interviews with parents of 11 children who had received an anxiety disorder diagnosis identified elements of the diagnostic process that were helpful or less helpful for families. In line with findings related to neurodevelopmental disorder diagnoses (e.g. Abbott et al., 2013; Ringer et al., 2020), receiving the diagnosis helped parents, children and others around them make sense of the child’s anxiety difficulties, and parents valued clinicians sharing information about their child’s anxiety disorder diagnosis sensitively. They also appreciated the opportunity to have a discussion about the diagnosis and to receive a comprehensive written report. Given that parents often report difficulties differentiating clinical levels of anxiety from developmentally appropriate fears and worries (Reardon et al., 2018), it is perhaps unsurprising that receiving clear and child-specific diagnostic information helped parents better understand the disorder and its manifestation in their child. Notably, it was the detailed description of their child’s anxiety difficulties, more than the diagnostic label itself, that seemed to enhance parents’ understanding and helped them feel more able to support their child. Indeed, parents who felt confident in their understanding of their child’s difficulties prior to the assessment viewed their child’s diagnosis as redundant and conveyed frustration towards the diagnostic process.

Parents’ experiences of the diagnostic process seemed to be shaped by their perceptions of the ‘anxiety label’, particularly the extent to which they felt it accurately captured their child’s difficulties and whether they had concerns about negative consequences associated with this label. It was notable that parents who expressed concerns about the accuracy of the diagnosis were parents of children who had received a primary diagnosis of GAD. Indeed, GAD is characterised by symptoms that overlap with other anxiety and depressive disorders, and arguably is a less coherently defined construct than disorders that are characterised by specific or situational fears (Reardon et al., 2019). Notably, concerns that the anxiety ‘label’ may result in a child being treated differently echo concerns related child mental health diagnoses more broadly (e.g. Eaton et al., 2017). Clinicians helped to alleviate these concerns about the ‘label’ by communicating optimism about the child’s future and sharing the diagnosis in the context of ensuing treatment. Consistent with the neurodevelopmental disorder literature (e.g. Makino et al., 2021), parents identified access to treatment as the key purpose of a formal diagnosis.

### Implications for clinical practice

The study findings have clear implications related to the use of child anxiety disorder diagnoses in clinical settings, how best to share outcomes from diagnostic assessments with families, and ways to minimise unintended negative consequences. Indeed, findings illustrate that using comprehensive diagnostic assessments in clinic settings and receiving anxiety disorder diagnoses can be a very helpful and positive experience for families. However, it is important to be transparent with families about the fact that some benefit from the diagnostic process more than others, for example by explaining that where families already feel confident in their understanding of their child’s difficulties, they may not gain further insight from the process. Clinicians should be clear about the time commitment involved, and clearly explain that this is needed to ensure accurate assessments and appropriate treatment recommendations. Where diagnoses are used, it is crucial that access to appropriate treatment and/or referrals to outside services follows.

Parents’ experiences clearly show that effective communication between clinicians and families throughout the diagnostic process, and ensuring families feel listened to and heard, are critical. In particular, findings highlight that when sharing diagnostic outcomes clinicians should (1) remain sensitive to the emotional impact of a diagnosis and communicate diagnoses compassionately in the context of ensuing treatment; (2) share personalised information that relates to the child’s *own* presentation; and (3) provide clear and tailored information for both parents and children (e.g. by sharing parent- and child-versions of the assessment report, using visual aids for children, talking to parents and children separately and together). It is also imperative to allow sufficient time to fully explain and discuss anxiety disorder diagnoses to aid a shared understanding of the problem. Sharing diagnoses verbally gives families an opportunity to ask questions, and our findings illustrate the benefits of also providing comprehensive written information for families and copies to share with others. Common concerns specifically related to anxiety disorders should also be addressed as part of the diagnostic process. For example, this study highlights the potential merit of clinicians reassuring families that child anxiety disorders are common and can be effectively treated. Indeed, discussions about diagnoses provide an opportunity for psychoeducation and allow families to make informed decisions about treatment options.

### Strength and limitations

Our rigorous, inductive qualitative approach provided detailed insight into the experiences of parents whose child had received an anxiety disorder diagnosis. However, it should be acknowledged that the lead researcher (ED) conducted the routine diagnostic assessment with some participants and this prior contact may have influenced what parents shared in the qualitative interview. Provisions were made to minimise researcher bias (e.g. ED kept a self-reflective journal), nevertheless the wider team’s experience in assessing and treating child anxiety disorders and general belief in the importance of consistent and thorough assessments inevitably influenced the collection and interpretation of the data.

It is also important to note that the sample was recruited from one UK child mental health service, and parents’ experiences of their child receiving an anxiety disorder diagnosis in other services and geographical settings may differ. We were unable to obtain complete sociodemographic data, although available data indicated that the sample was predominantly White British and exclusively mothers. Whilst this was reflective of the particular service, it will be critical that the views and experiences of families from different ethnic backgrounds, and experiences of fathers and other caregivers, are considered in future research. Differences in culture have a range of implications for mental health practice, including the meaning of health and illness, help-seeking patterns, and issues of racism and discrimination (Gopalkrishnan, 2018). It is, therefore, important that future studies capture the experiences of families from a wider range of services and backgrounds to gain insight into a broader spectrum of parents’ experiences. It would also be beneficial to explore adolescent perspectives on anxiety disorder diagnoses to extend the guidance for use of anxiety disorder diagnoses to this age group.

## Supplemental Material

sj-pdf-1-ccp-10.1177_13591045221088708 – Supplemental Material for ‘It opened my eyes’: Parents’ experiences of their child receiving an anxiety disorder diagnosis Click here for additional data file. Supplemental Material, sj-pdf-1-ccp-10.1177_13591045221088708 for ‘It opened my eyes’: Parents’ experiences of their child receiving an anxiety disorder diagnosis by Emily Davey, Cathy Creswell, Ray Percy and Tessa Reardon in Clinical Child Psychology and Psychiatry

sj-pdf-2-ccp-10.1177_13591045221088708 – Supplemental Material for ‘It opened my eyes’: Parents’ experiences of their child receiving an anxiety disorder diagnosis Click here for additional data file. Supplemental Material, sj-pdf-2-ccp-10.1177_13591045221088708 for ‘It opened my eyes’: Parents’ experiences of their child receiving an anxiety disorder diagnosis by Emily Davey, Cathy Creswell, Ray Percy and Tessa Reardon in Clinical Child Psychology and Psychiatry

sj-pdf-3-ccp-10.1177_13591045221088708 – Supplemental Material for ‘It opened my eyes’: Parents’ experiences of their child receiving an anxiety disorder diagnosis Click here for additional data file. Supplemental Material, sj-pdf-3-ccp-10.1177_13591045221088708 for ‘It opened my eyes’: Parents’ experiences of their child receiving an anxiety disorder diagnosis by Emily Davey, Cathy Creswell, Ray Percy and Tessa Reardon in Clinical Child Psychology and Psychiatry

## ORCID iD

Emily Davey https://orcid.org/0000-0001-5785-1533

Author Biography

Emily Davey is a PhD Student at UCL Great Ormond Street Institute of Child Health.Tessa Reardon is a Postdoctoral Researcher based at the University of Oxford.Ray Percy is a Principal Clinical Psychologist and the Director of the AnDY Research Clinic at the University of Reading.Cathy Creswell is a Professor of Developmental Clinical Psychology at the University of Oxford and an Honorary Consultant Clinical Psychologist in Oxford Health NHS Foundation Trust.
