# Supplementary material for: ‘It opened my eyes’: Parents’ experiences of their child receiving an anxiety disorder diagnosis
Source: Clin Child Psychol Psychiatry. 2022 Apr 25;27(3):658–69. doi: 10.1177/13591045221088708 (PMC9234767; doi:10.1177/13591045221088708)
Supplement: sj-pdf-2-ccp-10.1177_13591045221088708 – Supplemental Material for ‘It opened my eyes’: Parents’ experiences of their child receiving an anxiety disorder diagnosis [file sj-pdf-2-ccp-10.1177_13591045221088708.pdf]

Online Supplement 2: Details of Diagnostic Assessment

Each diagnostic assessment included separate child and parent interviews, administered by research assistants (Psychology graduates) trained to a high level of inter-rater reliability. The anxiety and behavioural sections of the Anxiety Disorders Interview Schedule for Children (ADIS-C/P; Silverman & Albano, 1996) were used to determine the presence of one or more anxiety disorder, and/or behavioural disorders. The depression and mania sections of the Kiddie Schedule for Affective Disorders and Schizophrenia (K-SADS; Kaufman et al., 1997) were administered to establish whether the child met diagnostic criteria for any depressive disorders. Minor amendments were made to the interview schedules to enable diagnoses congruent with DSM-5 diagnostic criteria. Diagnoses and clinical severity ratings (CSR) were assigned for each interview separately. In accordance with the guidelines, a child received a diagnosis when they met diagnostic criteria and a CSR from 4 to 8 was assigned. The disorder with the highest CSR was allocated as the child’s primary disorder.

References

Kaufman, J., Birmaher, B., Brent, D., Rao, U. M. A., Flynn, C., Moreci, P., Williamson, D., & Ryan, N. (1997). Schedule for Affective Disorders and Schizophrenia for School-Age Children-Present and Lifetime Version (K-SADS-PL): Initial Reliability and Validity Data. *Journal of the American Academy of Child & Adolescent Psychiatry*, *36*(7), 980-988. https://doi.org/https://doi.org/10.1097/00004583-199707000-00021

Silverman, W. K., & Albano, A. M. (1996). *The anxiety disorders interview schedule for DSM-IV-child and parent versions.* San Antonia, TX: Psychological Corporation.
