# Supplementary material for: ‘It opened my eyes’: Parents’ experiences of their child receiving an anxiety disorder diagnosis
Source: Clin Child Psychol Psychiatry. 2022 Apr 25;27(3):658–69. doi: 10.1177/13591045221088708 (PMC9234767; doi:10.1177/13591045221088708)
Supplement: sj-pdf-3-ccp-10.1177_13591045221088708 – Supplemental Material for ‘It opened my eyes’: Parents’ experiences of their child receiving an anxiety disorder diagnosis [file sj-pdf-3-ccp-10.1177_13591045221088708.pdf]

Supplementary Material 3: Topic Guide

Diagnostic process

Your child recently took part in a diagnostic assessment here in the clinic, and afterwards you came in for a follow up appointment and received a report in the post. I wonder if you could tell me a bit about how you found that process?

- *Probe:* How did you end up having a diagnostic assessment?
- *Probe:* Did you experience anything helpful/unhelpful in the diagnostic process?
  - Is there anything else that was helpful/unhelpful?
- *Probe:* What did you think of the follow-up appointment?
- *Probe:* What did you think of the written report?
- *Probe*: What did you think about the method in which the diagnosis was communicated to you?
  - Did the method of communication work?
- *Probe:* What improvements could be made to the diagnostic process?

Is there anything you would do differently if you were to go through this process again?

Response to the diagnosis

Have you spoken to your child about their diagnosis?

- *Probe:* How did you tell them about their diagnosis?
- *Probe:* How did they react when you told them?
- *Probe*: Did you notice any changes in your child since they were told?

If they have not told their child about their diagnosis:

- *Probe:* Was there any particular reason why you chose not to tell them?

How did you feel when your child was first diagnosed with (*dx*)?

- *Probe:* Were you expecting a diagnosis of (*dx*)?
- *Probe*: Did you feel you understood what the diagnosis meant?
- *Probe:* Did you feel the diagnosis was accurate?
- *Probe*: Does the diagnosis fit with your experiences?

How do you feel about your child's (*dx*) diagnosis now?

- *Probe:* Why do you think your feelings have changed?

What do you think the diagnosis means for your child's future?

- *Probe:* Do you perceive the diagnosis as long-term? Why do you think that is?

If the diagnosis has been revised/changed:

- *Probe:* How did you feel when your child's diagnosis was revised?

Do you feel that the diagnosis has altered the way you view/understand your child?

- *Probe:* Has anything changed in your family life since receiving the diagnosis?

Disclosing the diagnosis with others

Have you discussed your child's diagnosis with anyone else?
(Could you tell me a bit about that e.g., school, friends, family)

- *Probe:* How did you explain your child's anxiety to other people?
  - Did you tell them the (*dx*) diagnosis itself or just describe his/her difficulties?
- *Probe:* Was there a reason you decided to tell them?

- *Probe:* Was there anyone you were reluctant to tell?

How did people react when you told them?
- *Probe*: Has anything changed since telling them?
- *Probe:* How does this make you feel?

If they have not told other people about their child's diagnosis:
- *Probe:* Have you considered telling other people?
- *Probe*: Was there a particular reason that you did not want to tell them?

Relevance of anxiety diagnosis to family

Has the anxiety diagnosis helped your family? (e.g. giving meaning, seeking treatment)
- *Probe:* Do you think having the actual (*dx*) diagnosis has helped with that specifically?
- *Probe:* Do you think you could have had the same benefits without the diagnosis?

If child knows about diagnosis:
- Has the *(dx)* diagnosis helped your child?
    - In what ways?

Do you feel that the success of treatment has shaped the way you feel about the diagnosis?

Final questions

Do you have any last comments about your child's (*dx*) diagnosis?
